# Costing of Paediatric Treatment alongside Clinical Trials under Low Resource Constraint Environments: Cotrimoxazole and Antiretroviral Medications in Children Living with HIV/AIDS

**DOI:** 10.1155/2016/9456906

**Published:** 2016-11-29

**Authors:** Bona M. Chitah

**Affiliations:** Department of Economics, University of Zambia, Lusaka, Zambia

## Abstract

*Introduction*. Costing evidence is essential for policy makers for priority setting and resource allocation. It is in this context that the clinical trials of ARVs and cotrimoxazole provided a costing component to provide evidence for budgeting and resource needs alongside the clinical efficacy studies.* Methods*. A micro based costing approach was adopted, using case record forms for maintaining patient records. Costs for fixed assets were allocated based on the paediatric space. Medication and other resource costs were costed using the WHO/MSH Drug Price Indicators as well as procurement data where these were available.* Results*. The costs for cotrimoxazole and ARVs are significantly different. The average costs for human resources were US$22 and US$71 for physician costs and $1.3 and $16 for nursing costs while in-patient costs were $257 and $15 for the cotrimoxazole and ARV cohorts, respectively. Mean or average costs were $870 for the cotrimoxazole cohort and $218 for the ARV. The causal factors for the significant cost differences are attributable to the higher human resource time, higher infections of opportunistic conditions, and longer and higher frequency of hospitalisations, among others.

## 1. Introduction

The burden arising from the consequences of the spread of the human immune deficiency virus (HIV) and Acquired Immunodeficiency Syndrome (AIDS) epidemic in Zambia has been relatively high with respect to both morbidity and mortality effects. It has been estimated that the prevalence or progression of total infections of HIV increased from about 30,000 cases in 1993 to about 1,100,000 by 2010 while national population at this time was estimated to be 13 million [1–3]. It is currently estimated that the incidence or rate of new infections* per annum *is now slightly over 72,000 cases. This is an increase from about 67,000 cases* per annum* in 2006. The increase has been occurring in spite of a decline in adult prevalence (i.e., the proportion of sexually active adults between the ages of 15–49) from about 17 percent in 2000 to 14 percent in 2007. At the same time it has been estimated that of the 1,100,000 people living with HIV/AIDS (PLHA), a minimum of 490,000 are women while 120,000 are children. The annual AIDS-related mortality is in the region of 45,000 [4–7].

Apart from antiretroviral therapy, the World Health Organisation (WHO) has advocated the use of cotrimoxazole in low resource settings to enhance and complement ART administration. During the early part of 2000 decade, Medical Research Council (MRC) in collaboration with the University of Zambian Medical School undertook WHO supported clinical trials to evaluate clinical efficacy for initially cotrimoxazole and subsequently ARVs [2, 8].

Since the trials, the use of cotrimoxazole and ART for paediatrics has since been approved and is currently in use. However, the costs for treatment were not provided. Costing of the drugs, namely, cotrimoxazole and paediatric ARVs, became necessary in order to provide information on the cost-of-illness structure with respect to resource utilisation and programming budgets. Costing data plays an important aspect in the planning and budgeting processes of the Ministry of Health in Zambia which accounts for the provision of services to approximately 80% of the population [7]. This is very much a similar case in other low resource public health settings. In addition, it is just as useful set of information for the nongovernmental and private health providers as it serves identical needs in terms of assisting budgeting and planning functions. Budgeting and planning, undertaken under low resource constraints, allow institutions to prioritise and allocate resources as a means to balance service provision and delivery by facilitating or balancing resource needs to health needs. It serves as an important technical input into framing resource allocation based on resource use, costs, and their budgetary implications. This affords a balanced approach between resource needs based budgets as well as needs based budgets. The use of costing data, though crude, remains a second best option as a rational means for allocating resources.

This study augments the required cost data for children living with HIV/AIDS. This evidence is deemed essential or necessary in view of the lack of costing data on all major interventions available to the policy makers. For instance, it has been accepted that there needs to be a costed essential package of health care services that allows the health sector to have evidence of the cost of the interventions and their budgetary and resource allocation decisions [1]. In the absence of an essential package of health care which provides a costing of all essential interventions, the costing of ARVs, albeit for paediatric medications, does serve as an input into availing evidence and understanding the costing landscape of one of the leading public health concerns.

Research on costing of paediatric ARVs has been limited. A study by Marques et al. [9] focussed on costs relating to inpatient costs for children living with HIV/AIDS in a teaching hospital or tertiary setting in Brazil. Scott et al. [10] report on this in their study in outpatient costs of children on ART. They specifically cite two studies [11, 12] which have undertaken similar work. Iyer et al. [13] study resource utilisation and costs prior to ARV initiation which provides a different dimension of costs relating to HIV/AIDS care. Two other studies, Taiwo et al. [14] and Adesina and Waldron [15], assess the average costs of universal access to ART based on the Zambian 2013 HIV/AIDS Treatment Guidelines and incremental costs of providing paediatric ART. Doherty et al. [16] attempt to provide an analysis of age decomposition of ART costs for children in underresourced health systems as a means to providing an accurate representation of cost implications for general ART care. However, none of these studies have been able to provide simultaneous costing of two key medications for paediatric HIV/AIDS management as this study attempts to do, consequently presenting value for information to policy makers in view of the problems faced in planning and decision-making.

## 2. Materials and Methods

For purposes of the RCT, Pedimune comprising fixed dose combinations (FDCs) of nevirapine (NVP), stavudine (d4T), and lamivudine (3TC) was manufactured exclusively for purposes of facilitating the trials.  Table 1 outlines Pedimune composition.

The data collection and analysis methods built on the clinical trial process. A brief outline of each of these is provided below.

### 2.1. The Children with HIV Antibiotic Prophylaxis (Cotrimoxazole) Trial

The Children with HIV Antibiotic Prophylaxis (CHAP) trial was a phase III placebo controlled trial design in nature. Evaluating the prophylactic properties of cotrimoxazole in assisting with reducing morbidity and mortality of children living with HIV/AIDS served as the main purpose of the trial [17].

#### 2.1.1. Sample Size

The primary endpoint designated was time to death or first hospital admission after randomisation. The number of children assessed for eligibility was 1,185, of which 851 were tested for HIV antibodies. 699 children were determined to be HIV-antibody positive and of this 541 consented to be enrolled into the trial. These were subsequently randomised comprising 265 in the cotrimoxazole arm and 269 in the placebo arm.

### 2.2. The Children with HIV in Africa—Pharmacokinetics and Adherence of Simple Antiretroviral Regimes (CHAPAS) Trial

The main purpose and objective of the CHAPAS trial was to determine the efficacy of newly formulated three in one paediatric ARVs. Additional objectives included assessing pharmacokinetic and adherence characteristics and practices, respectively, of each of the medications [17, 18].

Eligibility for enrolment into the study was based on children with HIV aged between 3 months and 14 years, all inclusive, and who were eligible for ART, according to the WHO criteria.

#### 2.2.1. Sample Size

211 children were enrolled in a 1 : 1 ratio to start with Pedimune either at a full dose on a twice daily schedule or on a dose escalation schedule of once a day administration for 14 days which is subsequently scaled up to full dose, with the latter schedule having a 50 percent of normal dose of NVP. Enrolment took place over a 12-month period and follow-up continued until the last randomised child had completed a 48-week schedule.

#### 2.2.2. Outcomes

Primary endpoints were defined as either time to death or grade 3 or 4 adverse reactions. Secondary endpoints were determined as time to death or hospitalisation, episodes of definitive and presumptive bacterial infections, resistance to cotrimoxazole in children developing bacterial infections, development of* Streptococcus pneumoniae*, frequency of clinical malaria, changes in weight and height, and the number of courses of cotrimoxazole required.

### 2.3. Data Collection

#### 2.3.1. Clinical Data Collection: Case Report Forms

The data collection was made at the following points: (1) before trial commencement, (2) follow-up, that is, baseline and midline, and (3) end line of the trials. Data capture and recording used case report forms for daily capture of all resources used including care giving visits or facility visits by the patients and the carers. These forms captured both socioeconomic and clinical data. These data are summarised in Table 2.

Costing theory decomposes costs into different constituent parts. However, the application of cost theory seldom works in isolation as accounting methodologies and analytical tools are often, such as in the case of allocation of costs necessary. Allocation methodology, as shown in Table 2, for deriving relevant programme cost components arises due to the existence of global cost structures which cannot, by any feasible means, be decomposed into constituent unitary measures due to combined functional programmes and responsibility. Furthermore, we can derive unit costs assuming that the average long run costs divided by the output produce unit costs for the programme. HIV related complications are ultimately long term and their associated costs are correspondingly long term. Therefore, based on the fundamentals of cost theory, the derivation of long run average costs can in the absence of micro costing information be assumed to present fairly reliable cost estimates.

Costs are differentiated depending on their related activity or functionality. Patients, communities and society in general, and providers and policy makers, that is, ministries of health, may all have a different conceptualisation of costs and costs may be evaluated from different perspectives that include patient, provider, or societal [19]. Cost data was collected during the period of the trials and posttrial period. Resource use data was valued based on pricing of the inputs based on cost prices. These were either available through the procurement process or the* International Indicator of Drug Pricing* publication [20, 21].

#### 2.3.2. Data Analysis

Data analysis was done in Excel and Stata. The method for cost estimates and analysis in this study is based on the provider perspective.

Cost structures are determined on the basis of fixed and variable costs. The fixed costs include the investment costs such as infrastructure and administrative costs. Variable costs include costs associated with providing specific services. These may include drugs, medical supplies, and nursing care.

The cost estimates were adjusted to 2015 values using the United States consumer price indices for the period of 2006 and 2015. Administration costs such as the administration and management and security costs of the UTH were apportioned as a proportion of the share of the space of paediatric ward facilities of UTH. The laundry and catering costs were allocated based on the total inpatient load of the paediatric section relative to the total inpatient care admissions.

#### 2.3.3. Micro Costing Approach

Micro costing, alternatively termed as the “ingredients” approach, has been categorised as the most precise costing method and is associated as being the closest to a “gold standard” in costing terms [19]. This is because it is based on using the smallest unit of input or procedure and the related prices or costs at market or economic cost. For instance, in the case of drugs, the costs (prices) are based on either the unit consumption or dosage which is subsequently used to determine the cost of a course or treatment required.

#### 2.3.4. Costing of Overhead Items

A problematic issue in handling costs often arises in the case of shared costs under a hospital environment in which several services are offered. While there are direct programme or service costs related to a particular condition and set of patients, for example, medications, there are however complications with respect to the question of how shared services, such as space, administration, medical, and nursing care, should be apportioned. It has been noted that none of the few costing studies available on paediatric ART other than the study by Marques et al. [9] have addressed the question of shared resources and costs in a facility undertaking. Excluding shared costs may be quite valid, although it is recommended that some justification be made. Once excluded, results of the programme are presented as programmatic or institutional marginal contributions to total cost [22].

A direct allocation method has been used to apportion costs in the study. The application of this method is justifiable in spite of the implied or actual exclusion of interactions between or among other departments. However, given the nature of RCTs and that the bulk of overhead costs arose in the cases of laboratory diagnosis and inpatient costs, it is appropriate to adopt this method. Cognisance is also taken of the options that involve stepdown allocation or partial adjustments for interaction of overhead costs; stepdown allocation with iterations or full adjustment for interaction of overhead departments; or simultaneous allocation in which there is full adjustment for interaction of overhead departments [9, 22, 23].

#### 2.3.5. Cost Items and Resource Use

As the costs were collected alongside the clinical trials, the case report forms were used to derive the resource consumption units and utilised in the costing substudy.

The generic formula used in the costing exercise was as follows:

(1) HIV/AIDS  Costs  per  Treatment  Regimen=∑inLi+∑itKt+∑idDd+∑ilLl,

where *L*_*i*_ is labour costs for each particular cadre involved such as the medical doctor, nurses, and laboratory technicians. *K*_*t*_ is capital items involved including buildings, laboratory equipment, and motor vehicles. *D*_*d*_ represents medicines and medical supplies including ARVs, cotrimoxazole, and antibiotics. *L*_*l*_ is laboratory diagnostic tests for haematology, biochemistry, and lymphocyte and virology test.

#### 2.3.6. Resource Use Data

Data on resource use was collected throughout the entire duration of the study. The resources consumed during the study were identified on the basis of the structure of sources and types of resource consumption in Table 2.

The fixed cost items in the study were identified as laboratory equipment for laboratory analysis and performance of activities such as CD4 counts, HIV/AIDS testing, malaria testing, motor vehicles, and infrastructure for the health facilities.

The medications and medical supplies which were listed by individual patients were collected from the same sources as above.

#### 2.3.7. Building and Equipment Cost

The building costs are estimated as the percentage share of the space. The use of equipment presents an option for determining the equipment cost. Building and equipment costs were annuitised at 3%.

#### 2.3.8. Laboratory Costs

Laboratory costs were done for different tests. Among these were the following: stool culture, blood culture, sputum culture, urine microscopy HIV viral load, malaria parasite slide (MPS), cerebrospinal fluid (CSF) tuberculosis sputum, liver function tests, full blood count (FBC) differential tests, and T cell CD4 counts. These constituted some of the main and recurring tests which were captured for each individual and whose resource use was documented in terms of each test at a standard rate and unit costs were obtained from the WHO sources on pharmaceutical prices.

#### 2.3.9. Physician and Nursing Costs

The unit costs for physician consultation were based on the total number of visits made by the physician with respect to each patient. In the case of patients where death was the censoring event, the number of visits up to the time of death was computed. The data not captured as a result of death was treated as missing data. The missing data was averaged out [24].

The total monthly wage was divided by the amount of time spent on consultation or nursing. This share of costs was allocated to the patient and multiplied by the total number of visits.

#### 2.3.10. Medication Costs—ARVs and Opportunistic Infection Medications

The total consumption of medication was estimated. The unit costs of the medications used have been described above.

#### 2.3.11. Hospitalisation Costs

 An algorithm of hospitalisation costs is shown as follows:

Inpatient costs = direct + overhead costs
Costs = fixed costs + variable costs
Direct = costs of medications
Overhead = laboratory costs + catering costs + administration costs + security costs + building/infrastructure costs + transport costs + other costs
Laboratory costs = cost of sample tests per unit
Catering costs (“hotel”) = costs of meals per hospitalisation visit
Administration costs = apportioned costs per total visitation
Building costs = share of allocated costs to paediatric ARV care
Overhead = building + admin + security + security

The hospitalisation data was captured for each individual patient who might have been hospitalised. The length of stay (days hospitalised) was recorded for each patient along with all resource consumption. Cost estimates were computed on the basis of the unit costs and utilisation and estimated based on the total resource consumption and human resource costs. The inpatient fixed costs were based on the process of allocation previously mentioned. The hospital as a rule uses absorption costing for its management financial system. Consequently, shared costs are allocated using this approach which uses space and patient load as weighting criteria for allocation.

#### 2.3.12. Outpatient Costs

The outpatient costs comprise mainly the consumption of resources such as human resource, medications, laboratory reagents, and fixed costs relating to shared costs (infrastructure and administration). This approach was used to determine average costs. Average costs are a derivative of the unit costs and quantities of resources consumed.

#### 2.3.13. Hospital Administration Costs

The administration costs are disaggregated into hospital security, catering, laundry, infrastructure, and others. In each particular case, these are based on the space allocation of the paediatrics section as well as the clinical trials unit; the respective allocated costs were derived from the total paediatrics allocated costs of the University Teaching Hospital (UTH).

#### 2.3.14. Laboratory Costing and Other Medical Examinations

Laboratory costs were made on the following key requirements as a matter of routine and for the initial selection purposes. The laboratory tests undertaken were as follows:

- Haematology: the samples were tested for haemoglobin, mean cell volume (MCV), platelets, white cell count, neutrophil, and lymphocyte counts.
- Biochemistry: creatine, alanine aminotransferase (ALT), aspartate aminotransferase (AST), and bilirubin.
- Lymphocyte subsets: CD3, CD3+CD4, CD3+Cd8, and total lymphocyte count.

These tests, haematology (lymphocytes), biochemistry, immunology (CD4 count), and microbiology (culture), were conducted at the minimum at each nursing visit and/or when the respondent reported not feeling well.

## 3. Results

In the CHAP trial, the mean age of respondents at initiation was 66 months. The mean CD4 count and CD percent were 585 and 12%, respectively, with mean weight of 17 kg. This is shown in Table 3(a). The corresponding measures between 72 and 96 weeks following initiation for CD count, CD percent, and weight were 599, 14%, and 16 kg, respectively, and the recorded data at the end of the trial were 694, 14%, and 16 kg, respectively. During the two-year period the mortality for the CHAP cohort was almost 35% as 186 children died in total. Table 3(b) shows the select characteristics for the ARV cohort, the same as Table 3(a).

The ARV cohort descriptive data shows mean CD4 count at initiation of 550, CD4% of 13, and weight of 15 kg. Mean data at 72–96 weeks of trial shows CD4 count of 1050, CD4% of 29, and weight of 14 kg. End of trial data show CD4 count of 808, CD% of 20, and mean weight of 16 kg. The mortality rate for the ARV cohort was just under 10% as 22 children died out of the 211 initially recruited.

**Figure 1:**
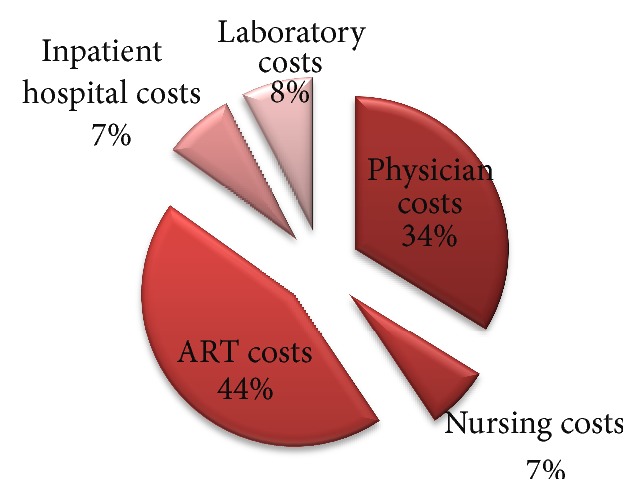
Disaggregated costs for the ARV cohort.

**Figure 2:**
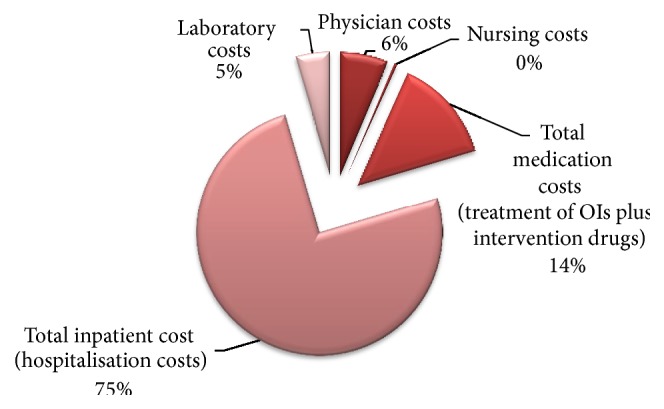
Disaggregated costs for the cotrimoxazole cohort.

The costs for the two cohorts differ significantly. Total physician costs were US$6,425 and the nursing costs were US$390 for the cotrimoxazole cohort. Similar costs for the ARV cohort were $15,240 and $3,080, respectively. The mean costs for nursing and physician costs are $1.3 and $16 for cotrimoxazole, while costs for the ARV cohort are $22 and $71, respectively. Other costs are shown in Table 4. Drug costs are further given in Tables 6 and 7. Figures 1 and 2 show the share of costs by function or item.

The differences in the costs can be distinguished by the different sources comprising mainly inpatient costs and treatment for opportunistic infections. In terms of outcomes, the height, weight, and, most importantly, the CD4% are relatively higher in the ART cohort.

The costs were determined based on the formulae provided earlier. Conceptually, these are summarised in Section 2.3.11 which outlines an algorithm of the costs and costing process. The different levels of costs comprise the outpatient and inpatient cost, human resources, and administration costs. These are inpatient costs that include laundry, security, catering, and utilities among the main ingredients of cost analysis. The other administrative costs such as amortisation costs were apportioned following the Ministry of Health practices within the UTH financial regulations and practices. These were allocated based on the space provided over 40 years for infrastructure such as buildings and for other assets such as motor vehicles these were done on a five-year period.

The main aim of the costing was not to provide an identically distributed set of costs but to capture a detailed unit reflective composition of costs consistent with the data collection method which was based on the collection of patient level data that uses individual resources consumed and valuation of these unit ingredients of resource utilisation.

The cost structure for the two therapies shows marked differences. The main drivers of the cost differences are hospitalisation frequency and occurrence of opportunistic infections. Hospitalisation in the case of the cotrimoxazole arm ranged up to 77 days as opposed to the ARV arm which had a maximum admission time of 37 days. Not only is the frequency much more in the case of cotrimoxazole, but also the seriousness of the illnesses and adverse events is much higher. This is again evident from the summary statistics presented in Tables 3(a) and 3(b).

**Figure 3:**
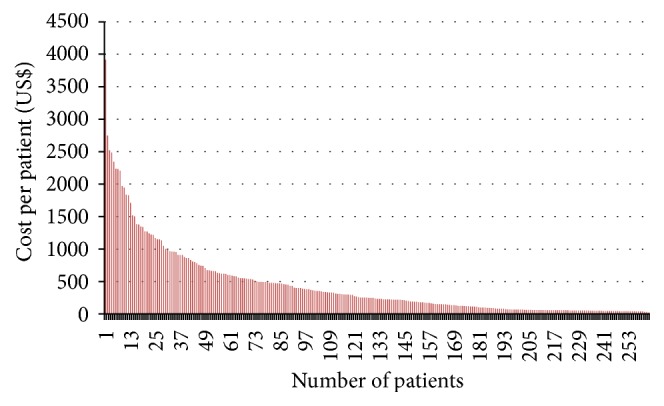
Cost distribution for the cotrimoxazole cohort.

**Figure 4:**
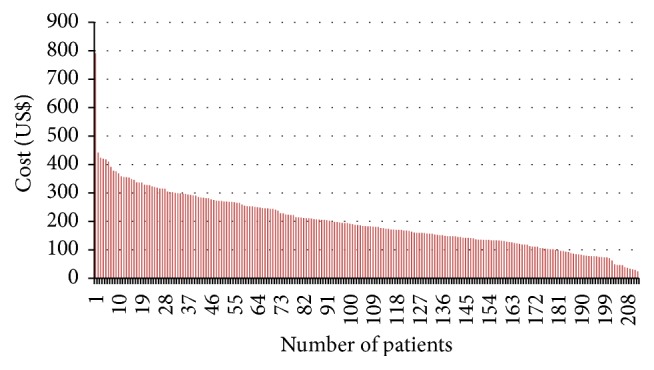
Cost distribution for ARV cohort.

The distribution of costs is skewed as may be expected with chronic conditions and relatively expensive therapies. Figures 3 and 4 show the cost data and the distribution effects of the extreme cases for the cotrimoxazole and ARV cohorts, respectively.

The skewness in the costs shown in Figures 3 and 4 is expected. Cost data typically have skewed distributions due to the minor proportion of patients who consume a disproportionate share of resources and consequently have a larger share of costs [25].

The structure of the costs for the two treatment arms shows quite diverse results. The costs for ARV are on the whole comparatively low. In particular the hospitalisation costs are markedly lower for ART than in the case of the costs for cotrimoxazole. The higher hospitalisation costs also reflect the frequency of hospitalisations and the higher costs related to the incidence of opportunistic infections. The cost structures ultimately have a key bearing on the final effectiveness results.

### 3.1. Opportunistic Infections

The recorded opportunistic infections and related drug therapy provided are shown in Tables 5(a) and 5(b). The OIs contributed significantly to the cost differential between the two cohorts. The cost differential is particularly acute when the hospitalisation is taken into account, in terms of both the duration (length of stay) and frequency of hospitalisation.

The causes of hospitalisation are also shown in Tables 5(a) and 5(b). Among the leading causes are diarrhoea, anaemia, fever, tuberculosis, malaria, and pneumonia. TB and pneumonia are particularly frequent for the cotrimoxazole cohort.

The cost structure shows a larger cost differential at the beginning in the first 1–5 years between the treatment care of comparator and intervention group. As the mortality rate is much higher for the cotrimoxazole group and much less for the ARV group, the cost differential narrows between the two groups as the surviving cases in the latter lead to higher relative treatment costs.

The pricing applicable for medication cost is provided in Tables 6 and 7 which show the unit costs for the various medications.

## 4. Discussion

While clinical trials are generally accepted as an ideal source of costing and cost evaluation data, there are nevertheless a number of concerns which are highlighted as possible areas of contention and caution. These concerns may be of relevance in the context of the two studies which are under consideration [22]:

- Data arising out of the need to meet the specific standards of the protocol different from routine generated data: protocols set out detailed and extensive monitoring and assessment criteria and data needs which may vary in practice; for example, the number of health care visits, nursing, physician care, and laboratory visits may be that much more extensive than under the normal environment. This may be further exacerbated by the rules and ethics concerning compliance, given the unknown consequences of the medication. These activities are most likely to lead to increased consumption use and increased costs.
- Censored data: this concerns not just the time of termination of the study but also the effect or consequences attributable to subjects that may be lost to follow-up or who simply drop out of the study prior to the attainment of the predetermined outcome. Again, the handling of these data gaps and subjects has to be decided regarding how it shall be managed in the analysis.

Cost analysis asks questions about the following: what type of cost information is needed, why such cost information is needed, or alternatively in whose interests are costs being collected and analysed? Related questions to these are how costs are then imputed from market and nonmarket resources and how overhead as well as capital outlays ought to be considered or managed. The underlying process in this study was to adopt methods treating costs from a micro perspective (“the ingredients” approach) constituting hospital care (utilisation/bed days; outpatient care and overheads) and community care comprising physician visits, nurse visits, and transport costs relating to clinicians' visits. Other costs related to direct patient care in terms of drugs/medications, laboratory costs, administrative costs (or other overhead costs), and equipment and building costs.

Issues relating to internal validity and generalisability are particularly anchored in the nature of the clinical trial as a mechanism for data generation. The randomisation process of the patients in the RCT is the basis of the assumption of the internal validity. The generalisability in the study is particularly emphasised by the choice of the alternative therapy, namely, cotrimoxazole, which in itself is of high policy relevance in terms of both domestic and international health.

The costs and cost analysis in the thesis are drawn from the resource use data from the randomised clinical trials. The total costs are computed as a function of the individual resource use from which the unit costs are derived. Allocations for the administrative costs were derived on the basis of the space use as well as time allocated to the care of the patients. Barber and Thompson [25] assert that when the costs of alternative treatment or interventions are required to guide health care policy decisions, what is important is the total cost for all the patients and, from this, the arithmetic mean. However, direct application of the cost data becomes problematic due to the skewed nature of the costs as shown earlier. The skewness is a consequence of the occurrence of extensive care needed by a few of the patients and the requirements of relatively costly care for some of the patients resulting in the concentration of costs around one part.

The distribution of costs and the range are consistent with the expected results. The results show almost a fourfold difference between the cotrimoxazole arm and the ARV arm. As mentioned earlier, although cotrimoxazole is cost-effective given low resource cost and being with no alternative, it is susceptible to exposure of the patients with more infections and a limited life expectancy.

The data show that the differences in the cost are quite considerable. The outcomes are also clearly given in terms of the deaths averted through the use of ARVs in relation to cotrimoxazole. In addition, the results show the differences in both cost differences and other intermediate outcomes such as the rate of hospitalisation, the frequency and rate of opportunistic infections, and/or the frequency of health facility utilisation. These are different endpoints which are different forms of outcome or effects.

The costing analysis is the first detailed analysis of costs for a paediatric ART programme based on clinical trials.

Cases of missing data did not occur as the data was being collected alongside the clinical trials. However, there were cases of censored events due to death. Data was collected up to the time of the event. There was in this regard a complete cycle of data collection. The limitation of censored events forms part of the skewness of the data which was addressed through the bootstrapping process referred to earlier and which can be further addressed through linear regression analysis [25].

Estimated cost results of cotrimoxazole and ART management show that there exist significant differences in using the two treatment interventions. Taking the key cost elements shows that the differences between cotrimoxazole and ART were as follows: physician costs were five times higher and nursing costs were almost 8 times higher while medications were almost twice as much for ART relative to cotrimoxazole, respectively. However, the hospitalisation costs and medications under hospitalisation times were almost four times as much for patients on cotrimoxazole patients on ART. The differences in total costs between the two therapies demonstrate the significance of the likelihood of the risk of misinterpretation of total cost alone as a determinant of cost savings in relation to HIV/AIDS. More fundamentally, the cost differences show that the costs will be higher due to the compounding or cumulative costs as patients fall increasingly susceptible to various illnesses with changes in CD4 counts over time due to decline in immunity. From both the provider and societal perspective, the resource demand due to these differences leads to loss of societal gains or benefits.

## Figures and Tables

**Table 1:** Pedimune composition for RCT.

Drug	Pedimune baby	Pedimune junior
Nevirapine	50 mg	100 mg
Stavudine	6 mg	12 mg
Lamivudine	30 mg	60 mg

Source: CHAPAS Final Protocol.

**Table 2:** Sources of resource consumption.

Type of resource	Source	Cost items	
		Costing unit	Costing procedure
Capital item	Buildings	Space	Allocation process of overhead costs
	Motor vehicle	Unit	Unit cost/allocation/time
	Laboratory equipment	Unit	Price of equipment

Recurrent (direct costs)	Primary medications	ART drugs	Unit costs
	Medications for adverse events and opportunistic infections	Drugs	Unit costs

Human resource	Administration	Time of service provision	Allocation of time factor (hours) of total cost
	Medical doctor	Time of service provision	Allocation of time factor (hours) of total cost
	Nurses	Time of service provision	Allocation of time factor (hours) of total cost
	Laboratory technicians	Time of service provision	Allocation of time factor (hours) of total cost
	Laboratory reagents		

Overhead costs	Bed space	Cost per day	Allocation by share of capacity of paediatric section and estimated ARV patient load

Laundry			
Catering	Meals	Cost of catering	Allocation by share of capacity of paediatric section and estimated ARV patient load
Utilities	Electricity	Cost	
	Water	Cost	Allocation by share of capacity of paediatric section
	Security	Cost	Allocation by share of capacity of paediatric section

**(a):** 

	Age (months)	CD4 count	CD4 percent	Weight (kg)
Descriptive statistics at commencement of CHAP trial				
Mean	66	585	12	17
Median	57	449	11	16
Range	178	5422	54	38
Minimum	0	4	0	2
Maximum	178	5426	55	39

Progression of descriptive statistics at 48–72 weeks after initiation of cotrimoxazole				
Mean	73	599	14	16
Median	64	601	13	14
Range	177	1813	58	33
Minimum	0	22	0	6
Maximum	177	1835	58	39

Descriptive statistics at commencement of CHAP trial				
Mean	46	694	14	13
Median	38	587	13	11
Range	173	3288	56	27
Minimum	6	2	0	4
Maximum	179	3290	57	31

**(b):** 

	Weight (kg)	Age (months)	CD4 count	CD4 percent
Descriptive statistics at commencement of CHAPAS trial				
Mean	15	6	550	13
Median	15	5.7	375	12
Range	22.8	14.7	3700	38
Minimum	3.2	0.2	2	0
Maximum	26	14.9	3702	38

Descriptive statistics at 72–96 weeks of ARV initiation in CHAPAS trial				
Mean	14	7	1050	29
Median	14	7	907	29
Range	23	15	3819	67
Minimum	3	0	17	2
Maximum	27	15	3836	69

Descriptive statistics at end of CHAPAS trial				
Mean	16	6	808	20
Median	16	7	766	19
Range	17	14	2265	48
Minimum	8	1	25	1
Maximum	24	15	2290	49

**Table 4:** Average costs for cotrimoxazole and ARV medications.

	ARV				Cotrimoxazole			
Variable	Mean costs (US$)	Standard deviation	Minimum	Maximum	Mean (US$)	Standard deviation	Minimum	Maximum
Physician costs	71	21.8	6	107	22	6.8	3.6	32.4
Nursing costs	16	4.4	1	23	1.3	0.56	1	2
ART and cotrimoxazole costs (including other drugs for outpatient prescriptions)	91	70.9	0	307	45.6	132.8	0	2110
Total inpatient cost (hospitalisation costs including drugs)	13.4	32.6	0	516	250	446.45	0	3900
Outpatient costs	46		0	2110	46		0	2110
Laboratory costs	15	0	15	15	15	0	15	15
Total unit costs per person	218	95.2	24	798	871	556	1	4290

**(a):** 

	Frequency	Percent
Abscess/diarrhoea	1	0.26
Abscess/pneumonia	1	0.26
Anaemia	2	0.52
Anaemia/TB	2	0.52
Bronchitis/empyema	1	0.26
Bronchitis/pneumonia	1	0.26
Cellulitis pneumonia	1	0.26
Chicken pox	3	0.78
Conjunctivitis/marasmus/diarrhoea	1	0.26
Diarrhoea	12	3.1
Diarrhoea/abscess/TB/bronchitis	3	0.78
Diarrhoea/kwashiorkor/paronychia	2	0.52
Diarrhoea/lymphadenopathy	1	0.26
Diarrhoea/malaria	1	0.26
Diarrhoea/pneumonia/malaria/lymphadenopathy	1	0.26
Diarrhoea/pneumonia/marasmus/kwashiorkor/TB	10	2.6
Diarrhoea/TB	4	1.04
Eczema/pneumonia	1	0.26
Fevers/measles/TB/pneumonia	4	1.04
Gastroenteritis/dysentery/gastritis	7	1.82
Herpes simplex	1	0.26
Herpes zoster/pneumonia	3	0.78
Lymphadenopathy/parotitis	5	1.3
Malaria	9	2.33
Malaria/anaemia/diarrhoea/malnutrition/TB/marasmus/meningitis/pneumonia	18	4.68
Malnutrition/TB/measles/marasmus/chicken pox/diarrhoea	17	4.42
Marasmus/kwashiorkor	2	0.52
Marasmus/kwashiorkor/diarrhoea/dehydration	1	0.26
Marasmus/kwashiorkor/pneumonia/TB	10	2.6
Measles	4	1.03
Measles/pneumonia	2	0.52
Meningitis/malaria/pneumonia/otitis media pneumonia	15	3.89
Pleural effusion	1	0.26
Pneumonia	49	12.74
Pneumonia/abscess/malaria/TB/fever/kwashiokor/measles/ari/septecemia	27	7.02
Pneumonia/anaemia	44	11.44
RTI	20	5.2
Septic arthritis	1	0.26
Septicaemia	1	0.26
Septicaemia/meningitis	1	0.26
Septicaemia/meningitis/pneumonia/TB	1	0.26
TB	75	19.5
TB/diarrhoea/malnutrition/thrush/meningitis/neutritis/tonsilities	15	3.9
Unknown	3	0.78
Chicken pox	1	0.26
UTI	1	0.26
UTI/oral thrush	1	0.26

**(b):** 

Condition	Frequency	Percent
Papular pruritic eruptions	1	0.13
Extensive molluscum contagiosum	1	0.13
Recurrent oral ulcerations (2 or more episodes in 6 months)	1	0.13
Unexplained persistent parotid enlargement	2	0.27
Recurrent or chronic upper respiratory tract infections (otitis media, otorrhoea, sinusitis, tonsillitis)	1	0.13
Moderate unexplained malnutrition not adequately responding to standard therapy	83	11.05
Unexplained persistent diarrhoea (>14 days)	163	21.7
Unexplained persistent fever (above 37.5 intermittent or constant, for >1 month)	176	23.44
Persistent oral candidiasis (outside neonatal period)	74	9.85
Oral hairy leukoplakia	1	0.13
Acute necrotizing ulcerative gingivitis/periodontitis	1	0.13
Pulmonary tuberculosis	70	9.32
Severe recurrent presumed bacterial pneumonia	79	10.52
Unexplained anaemia (<8 gm/dL), neutropenia (<500/mm^3^), or thrombocytopenia (<50,000/mm^3^) for > 1 month	10	1.33
Symptomatic lymphoid interstitial pneumonia (LIP)	4	0.53
TB lymphadenitis	1	0.13
Unexplained severe wasting or severe malnutrition not adequately responding to therapy	72	9.59
Recurrent severe presumed bacterial infections	1	0.13
Chronic herpes simplex infection (orolabial or cutaneous, for >1 month, visceral at any site)	1	0.13
Extrapulmonary TB	5	0.67
Kaposi's sarcoma	2	0.27
Oesophageal candidiasis (or candida of trachea, bronchi, or lungs)	1	0.13
Extrapulmonary cryptococcosis including meningitis	1	0.13

**Table 6:** Unit prices of ARVs (dosage).

ARVs	Dosage	ARV prices (US$)
Lamivir Baby	0.3 g	0.0313
Lamivir Junior	0.3 g	0.0587
Efavirenz	0.6 g	0.154
Pedimune Baby	0.6 g	0.023
Pedimune Junior	0.8 g	0.006
Triomune	0.3 g	0.058
Lamivudine	0.3 g	0.006
Stavudine	0.8 g	0.023

**Table 7:** Unit Prices of cotrimoxazole prophylaxis and opportunistic infection medication (dosage).

Drug	Dosage
Acyclovir	0.0468
Amoxycillin	0.0057
Ampicillin	0.098
Benzyl Penicillin	0.022716
Brufen	0.0042
Ceftriaxone	0.5156
Cephalexin	0.0468
Cetirizine	0.018
Chlorpheniramine	0.0017
Chloramphenicol	0.45
Ciprofloxacin	0.0257
Cloxacillin	0.0551
Coartem	0.0268
Cotrimoxazole	0.2
Erythromycin	0.0532
Augmentin	0.687
Fansidar	0.0173
Ferrous sulphate	0.0052
Flagyl	0.0395
Fluconazole	0.1455
Folic acid	0.0024
Frusemide	0.0494
Gentamycin	0.0024
Griseofulvin	0.0042
Isoniazid	0.1032
Ketoconazole	0.0024
Mebendazole	0.0178
Nalidixic acid	0.0438
Panadol	0.0024
Paracetamol	0.0255
Penicillin V	0.0071
Piriton	0.0017
Prednisolone	0.0762
Procaine penicillin	0.822
Promethazine	0.0061
Pyrazinamide	0.1774
Quinine	0.1032
Rifampicin	0.1032
Salbutamol	0.0045
Xpen	0.255
